# Cellular Plasticity and EMT in Cancer and Fibrotic Diseases

**DOI:** 10.3390/ijms27188355

**Published:** 2026-09-19

**Authors:** Margherita Sisto, Sabrina Lisi

**Affiliations:** Section of Human Anatomy and Histology, Department of Translational Biomedicine and Neuroscience (DiBraiN), University of Bari “Aldo Moro”, Piazza Giulio Cesare 1, I-70124 Bari, Italy; sabrina.lisi@uniba.it

Epithelial–mesenchymal transition (EMT) is a dynamic differentiation process during which epithelial cells transition toward a mesenchymal phenotype. This transformation is accompanied by enhanced motility, invasive potential, heightened resistance to programmed cell death, stemness traits, and an elevated deposition of extracellular matrix (ECM) components. In addition to physiological roles, EMT drives diverse pathological conditions, including progressive organ fibrosis, chronic autoimmune disorders, and oncogenesis. The current scientific consensus highlights EMT as a highly flexible process, with cells exhibiting hybrid states that span the epithelial–mesenchymal spectrum. This phenomenon, known as epithelial–mesenchymal plasticity (EMP), has sparked intensive research into how these fluid cellular states orchestrate tissue fibrosis, malignant progression, and metastatic dissemination.

This Special Issue aimed to establish a forum to highlight the newly identified molecular mechanisms involved in EMT/EMP that are linked to fibrotic diseases, autoimmune diseases, and cancer, providing the readers with a comprehensive overview of the current and emerging research in this field. As a result, this Special Issue collected eight high-quality articles represented by four literature reviews, one brief report, and three original research papers, spanning diverse research perspectives while remaining strictly aligned with the core theme.

A pivotal area of investigation since the mid-2010s has been the characterization of inflammasomes, complex multiprotein complexes that play a fundamental role in governing inflammation and innate immunity. These inflammasomes are platforms that are assembled and activated in response to pathogen-associated molecular patterns or DNA fragments released upon cellular damage. Inflammasome activation triggers a cascade of events, including the activation of caspase-1, the secretion of proinflammatory cytokines, and cell death through apoptosis or pyroptosis. Because aberrant inflammasome activation underlies various autoimmune conditions, fibrotic diseases, and malignancies, extensive research is now directed toward identifying the mechanisms driving this pathology for developing novel therapeutic interventions.

In this context, Sisto and Lisi explored the involvement of two specific inflammasomes, NLR family pyrin domain-containing 3 (NLRP3) and absent in melanoma 2 (AIM2), in autoimmune Sjögren’s disease (SjD). The aberrant activation of NLRP3 and AIM2 is implicated in fueling chronic inflammation, which induces ocular and oral dryness and elevates the risk of B-cell non-Hodgkin’s lymphoma in these patients. Ultimately, these findings contribute to a clearer understanding of the interconnected inflammatory and fibrotic processes, offering a promising approach for the discovery of novel therapeutic targets to counteract fibrogenesis (Contribution 1).

In their literature review, Bastos et al. outline how cellular plasticity allows cells to dynamically adapt their phenotype to environmental cues, thereby driving tumor progression and fibrotic remodeling. The authors integrate recent insights into the molecular regulation of EMT, spanning signaling pathways (TGF-β, WNT, NOTCH, and HIPPO), transcription factors (SNAIL, ZEB, and TWIST), microRNAs, and epigenetic modifications. They highlight shared mechanisms and disease-specific features, underscoring the translational potential of targeting EMT plasticity. Furthermore, they discuss how emerging technologies such as single-cell transcriptomics and lineage tracing are advancing our understanding of EMT in diverse pathologies (Contribution 2).

Saima Ghafoor et al. conducted a comprehensive review, synthesizing the recently accumulated data on how EMT drives epithelial cancer progression, highlighting the intricate interplay and convergence of the multiple signaling pathways and transcription factors that regulate this complex process (Contribution 3).

In their study, Valentina Lopardo et al. designed an in vitro cell model to investigate glioblastoma-associated fibrotic alterations. Although cancer-associated fibrosis is a central process in extracranial cancer pathophysiology, its role as a tumor-promoting or tumor-restraining factor in glioblastoma remains to be fully elucidated. Because radiotherapy and cytotoxic chemotherapy trigger EMT and amplify TGF-β signaling, the authors investigated TGF-β1-induced alterations in the U-87MG FIBRO cell line. These changes significantly increased temozolomide (TMZ) resistance while altering cytotoxicity, senescence, and apoptosis, in agreement with earlier findings that fibrotic-like features promote tumor cell survival (Contribution 4).

Ovarian cancer (OC) remains one of the most lethal gynecologic malignancies, with nearly 80% of patients diagnosed at advanced stages due to the absence of early symptoms and the nonspecific nature of later clinical manifestations. This aggressiveness is largely driven by the EMT process, the dysregulation of which is widespread in OC and fuels tumor heterogeneity, metastatic spread, and chemoresistance. In their study, Matteo Cassandri et al. investigated the role of EMT-related genes in OC biology, analyzing the whole-genome sequencing and RNA-seq data from 419 patients to assess their genomic and transcriptomic alterations. They revealed recurrent alterations across several EMT-associated genes, suggesting that EMT-driven molecular changes contribute to the onset and progression of OC while highlighting a subset of EMT genes as promising predictive biomarkers for targeted therapy responses (Contribution 5).

Hepatocellular carcinoma (HCC) is an aggressive metastatic cancer with a high mortality rate, necessitating a deeper understanding of the molecular pathways regulating tumor cell states. To map these dynamics, Yıldız et al. used an LZTR1-knockout (KO) Hep3B model to characterize the consequences of losing this key negative regulator of oncogenic signaling. Their findings reveal that complete LZTR1 deficiency robustly upregulates phosphorylation across the RAF/MEK/ERK/RSK axis. Concurrently, LZTR1 loss induces a profound suppression of vimentin expression at the mRNA and protein levels. Given the pivotal role of vimentin in EMT, this downregulation directly accounts for the attenuated migration and invasion of LZTR1-KO cells. Taken together, these results characterize a novel EMT-related molecular and phenotypic state driven by LZTR1 deficiency, elucidating how the loss of LZTR1 structurally alters tumor cell behavior in HCC (Contribution 6).

Endothelial-to-mesenchymal transition (EndMT) represents another facet of EMT associated with cardiovascular, inflammatory, and fibrotic disorders, alongside neoplasia. Recent research tracking the COVID-19 pandemic shows that the SARS-CoV-2 peptide fragments can induce EndMT. Investigating this pathway, Barilli et al. found that cytokines secreted upon Spike S1 protein exposure drive phenotypic shifts in human umbilical vein endothelial cells (HUVECs), forcing the acquisition of mesenchymal markers including N-cadherin and fibroblast-specific protein 1 (FSP1). These results confirm that COVID-19 cytokine storm directly fuels endothelial dysfunction and downstream fibrosis, highlighting infliximab and baricitinib as clinically relevant strategies for preventing vascular damage (Contribution 7).

Snail and Zeb1 expression mark a hybrid/mixed epithelial/mesenchymal (E/M) state during EMT. Cancer cells in this intermediate state coexpress E- and M-specific transcripts and harbor cancer stem cell properties. This coexpression of Snail and Zeb1 was notably exhibited by M13HS-2/-8 tumor hybrid clones, derived from fusing human M13SV1-EGFP-Neo breast epithelial and HS578T-Hyg breast cancer cells. Addressing this mechanism, Keil et al. investigated the role of Snail across the E, E/M, and M states of these cell lines and their M13HS hybrids. They found that the elevated invasive capacity of M13HS-2 and M13HS-8 clones underscores how tumor hybridization yields altered cellular properties, such as enhanced metastatic potential or chemoresistance (Contribution 8).

In conclusion, this overview of the high-quality articles effectively encapsulates the current understanding of cellular plasticity and EMT in cancer and fibrotic diseases. By highlighting how dynamic cellular remodeling drives malignant progression and pathological tissue fibrosis, these findings underscore the universal role of plasticity in disease evolution. Determining the precise molecular mechanisms that control the EMT process confirms that targeting these transitional pathways remains a key frontier in modern medicine.

## Data Availability

The data presented in the special issue derive from a meticulous analysis of publications indexed on PubMed.

